# Towards a Risk-Based Follow-Up Surveillance Imaging Schedule for Children and Adolescents with Low-Grade Glioma

**DOI:** 10.3390/curroncol31110541

**Published:** 2024-11-18

**Authors:** Kleoniki Roka, Karina J. Kersbergen, Antoinette Y. N. Schouten-van Meeteren, Shivaram Avula, Astrid Sehested, Maria Otth, Katrin Scheinemann

**Affiliations:** 1 Division of Pediatric Hematology-Oncology, First Department of Pediatrics, National and Kapodistrian University of Athens, “Aghia Sophia” Children’s Hospital, 11527 Athens, Greece; 2 Department Neuro-Oncology, Princess Máxima Center for Pediatric Oncology, Heidelberglaan 25, 3584 CS Utrecht, The Netherlands; a.y.n.schouten@prinsesmaximacentrum.nl; 3 Department of Radiology, Alder Hey Children’s Hospital NHS Foundation Trust, Liverpool L14 5AB, UK; shivaram.avula@alderhey.nhs.uk; 4 Department of Pediatrics and Adolescent Medicine, Copenhagen University Hospital Rigshospitalet, Blegdamsvej 9, 2100 Copenhagen, Denmark; astrid.marie.sehested@regionh.dk; 5 Division of Oncology-Haematology, Children’s Hospital of Eastern Switzerland, Claudiusstrasse 6, 9006 St. Gallen, Switzerland; maria.otth@ksipisg.ch (M.O.); katrin.scheineman@kispisg.ch (K.S.); 6 Department of Oncology, University Children’s Hospital Zurich, Lenggstrasse 30, 8008 Zurich, Switzerland; 7 Faculty of Health Sciences and Medicine, University of Lucerne, Alpenquai 4, 6005 Lucerne, Switzerland

**Keywords:** low-grade glioma, pilocytic astrocytoma, cerebellar, surveillance imaging, imaging follow-up, children, adolescents, progression, relapse

## Abstract

The frequency and duration of imaging surveillance in children and adolescents with pediatric low-grade gliomas (pLGGs) aims for the early detection of recurrence or progression. Although surveillance of pLGGs is performed routinely, it is not yet standardized. The aim of the current review is to provide a comprehensive synthesis of published studies regarding the optimal frequency, intervals, and duration of surveillance. Several key influencing factors were identified (age, the extent of resection, the tumor location, the histological type, and specific molecular characteristics). However, the lack of consistent definitions of recurrence/progression and the extent of resection meant that it was not possible to perform a meta-analysis of the data from the 18 included articles. This review highlights the need for updating the definition of these terms for uniform and global use both in routine clinical practice as well as in upcoming trials. Thus, future studies on the heterogenous group of pLGGs will allow for the better tailoring of both the frequency and duration of imaging surveillance protocols in relevant settings.

## 1. Introduction

Low-grade gliomas (LGGs) are the most common type of brain tumors in children and adolescents, accounting for almost 30–40% [1,2]. Despite an excellent prognosis with a 20-year overall survival (OS) exceeding 87% [3,4], patients with pediatric LGGs (pLGGs) frequently relapse or progress with progression-free survival (PFS) rates of 30–40% [5], and may often suffer chronic neurological, visual, cognitive, and/or functional complications. These complications result from brain injury caused either by the tumor, the treatment, or a combination of both [6]. PLGGs are therefore considered a chronic disease in a significant proportion of patients [7].

The increasing understanding of the molecular landscape of pLGGs has improved our perception of their heterogeneity due to different epidemiological, clinical, radiological, histological, and molecular characteristics, as well as the variability in behavior and treatment responses. Furthermore, the recent study of dabrafenib–trametinib for patients with primary non-resectable pLGGs and BRAFV600 mutations which resulted in significantly more responses and longer PFS compared to standard chemotherapy (carboplatin plus vincristine) [8], alongside the promising results of tovorafenib in progressive LGGs [9] leading to an ongoing phase 3 randomized trial comparing tovorafenib versus standard-of-care chemotherapy in primary LGGs (LOGGIC/FIREFLY-2 trial) [10], imply that we are entering a new treatment era.

Despite the enormous evolution of knowledge in molecular and imaging techniques, there is a lack of evidence regarding optimal surveillance imaging in patients with pLGGs. To our knowledge, only two studies have been published in 1994 and 2019 that include staging and surveillance recommendations and take parameters such as the tumor growth rate, location, and patterns of local and metastatic recurrence for pLGGs into account [11,12]. In addition, even though several risk factors for poor prognosis have been identified, i.e., younger age, incomplete resection, midline location, histology other than pilocytic astrocytoma, specific molecular alterations (i.e., BRAFV600E), the presence of metastasis, and irradiation [3,4,5,13,14,15,16], the imaging follow-up recommendations do not take these into account. Moreover given the known risk of gadolinium deposition and renal toxicity in children [17], the possible effects of general anesthesia on younger children needing sedation for Magnetic Resonance Imaging (MRI) [18,19], as well as the psychosocial burden caused in patients and their families by repetitive MRIs, the need for rationalized surveillance imaging based on current knowledge is becoming more and more imperative.

This systematic review aims to provide a comprehensive synthesis of published studies examining the frequency, intervals, and duration of surveillance imaging to detect relapse or disease progression in survivors with pLGGs and identify specific subgroups of pLGGs requiring less or more frequent follow-up.

## 2. Materials and Methods

We conducted a systematic literature search according to the PRISMA guidelines in PubMed in January 2024 [20]. The search strategy used the following concepts: “low-grade gliomas” for the disease, “children, adolescents, and young adults” for the population, imaging by “Magnetic Resonance Imaging” as the intervention, and “surveillance, follow-up”, “progression, relapse” (see Supplementary Data S1 for the full terms). The search strategy was restricted to studies published between 1 January 2003 and 31 December 2023 and publications with animal models were excluded. Case reports and small case series with less than 5 patients were also excluded. The inclusion criteria were given through the PICO framework [20]. The population included children, adolescents, and young adults younger than 25 years at diagnosis or a maximum median age of 30 years of the whole cohort at diagnosis, and nearly all had a tissue-confirmed pediatric-type LGG. The maximum median age of 30 years of the whole cohort at diagnosis aimed to include studies with mixed-age populations, with older patients being included only if diagnosed with a pLGG. If identifiable, only the patients < 25 years were included in the analysis. The intervention corresponded to the frequency of surveillance imaging with MRI in survivors with pLGGs regarding age, location, histological type, the extent of resection, and relapse or progression of the tumor. We aimed to identify specific subgroups of patients with pLGGs that might need less or more frequent imaging. We also searched for secondary outcomes, including malignant transformation. The final reporting of the secondary outcomes depended on the provision of these data in the eligible publications.

Data from the eligible studies were extracted onto a standard data sheet, including information such as the first author, the year of publication, the study design, the treatment era and years of follow-up, patient characteristics and histological diagnosis, the imaging surveillance protocol if available, and the main and secondary outcomes. References within the eligible studies were additionally checked for possible additional publications fulfilling the inclusion criteria. Finally, publications with overlapping populations were included in the review and their data were extracted while critically determining the potential overlap.

The protocol for this review was published on Prospero (https://www.crd.york.ac.uk/prospero (registration date: 10 June 2024); ID: CRD42024551516).

## 3. Results

### 3.1. Description of Studies

**Figure 1:**
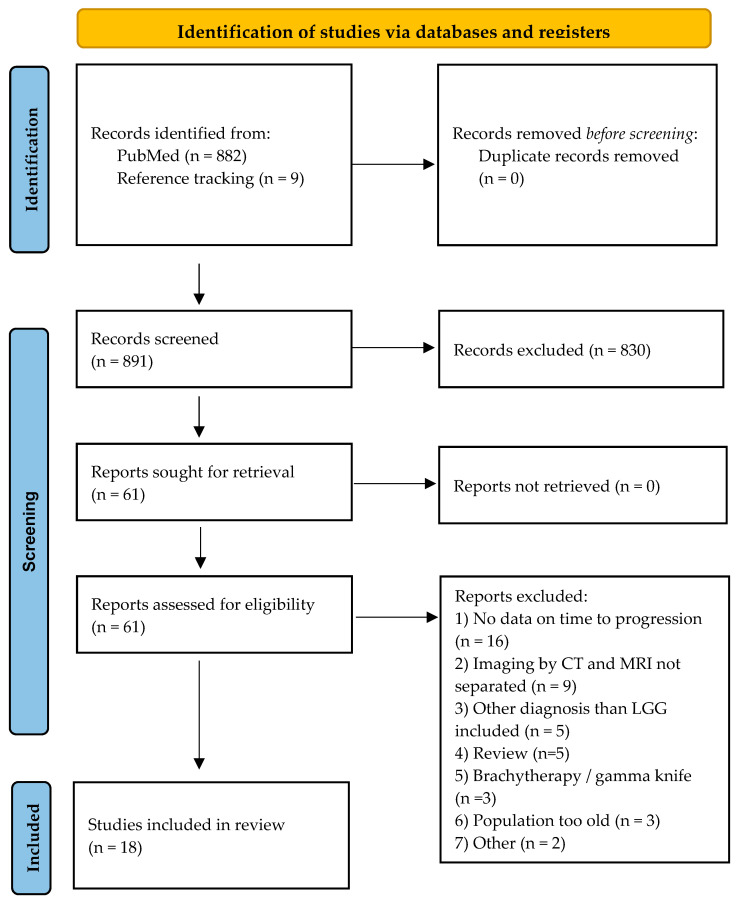
PRISMA flow diagram [38]. For more information, visit: http://www.prisma-statement.org/ (accessed on 8 September 2024).

The literature search identified 882 publications. A total of 56 potentially relevant full-text articles were retrieved for further evaluation. Thirteen publications met the inclusion criteria during the full-text screening. Reference screening of these 13 publications resulted in nine additional potentially eligible publications, of which five fulfilled the inclusion criteria. We finally included 18 publications in this systematic review (PRISMA flow diagram, Figure 1). There were two multicenter [5,21] and one prospective single-center study [22]. Thirteen studies were published after 2010, with five published since 2019 [21,22,23,24,25]. Six studies were carried out in Europe [21,22,23,25,26,27], seven in the USA [24,28,29,30,31,32,33], three in Canada [15,34,35], one in Australia [36], and one in the Republic of Korea [37]. The design of the studies as well as the heterogeneity of the reported outcomes allowed a descriptive analysis of the data only. The total number of patients with pLGGs was 3188, with a median sample size of 60 per study (range: 19–1031). It should be underlined that it was impossible to discriminate and remove double cases from the overlapping patients and periods. In the 15 studies where biological sex was defined, males represented 52.2% (1171 out of 2244 patients). Neurofibromatosis type 1 (NF1) was reported in 13 studies with 242 patients included, mainly with non-tissue confirmed pLGGs. Patients with Tuberous Sclerosis (TSC, 17 patients) were reported in two studies [5,25] and included without separate reports in one study. No other cancer predisposition syndromes were mentioned.

Data from three publications were either included [29,35] or overlapped [30] with another three publications, respectively [15,24,33]. Since we could perform a descriptive analysis of the data only and different/further information was provided in these publications, all six publications were analyzed. In particular, Tibbetts et al. analyzed the histopathological predictors for pilocytic astrocytoma (PA) event-free survival (EFS) [33] and overlapped with Dorward et al., which reported on imaging surveillance in PAs [29]. Kim et al. reported on a smaller number of patients regarding recurrence after gross-total resection (GTR) [30], whereas Zaazoue et al. studied a larger number of patients and focused on optimal postoperative surveillance [24]. Finally, Nolan et al. focused on long-term outcomes in patients with dysembryoplastic neuroepithelial tumors and included patients until 2002 [35], whereas Ryall et al. reported the clinical and molecular characteristics of 1000 pLGGs in 2020 [15].

#### 3.1.1. Definitions

Definitions of the extent of resection (Table 1) and progression or relapse (Table 2) were given in detail in 13 and 10 studies, respectively, and showed considerable variation. In only one study was the extent of resection based on the neurosurgeon’s macroscopically judgment [37]; in three studies combined and in 14 others, it was determined by radiological findings (Table 1).

#### 3.1.2. Description of Studies According to the Location of the Tumor

Nine out of the 18 studies included pLGGs irrespective of location [5,15,24,25,30,31,33,35,36], and in eight studies, the tumor was exclusively infratentorial or cerebellar [23,26,27,28,29,32,34,37]. One study analyzed only thalamic or thalamopeduncular LGGs [22]. Recurrence or progression of the tumor was reported in 28.4% of the patients with pLGGs in all studies analyzed (908 out of 3188 pts), with a median value of 20.4% (range 4.5–44.4%), whereas in the studies with only cerebellar/infratentorial tumors, the median progression/recurrence rate was 17.3% (range 7.1–38.4%) [23,26,27,28,29,32,34,37].

Ryall et al. reported a significant association between tumor location, PFS, and OS with the best 10-year PFS and OS for tumors located in the cerebellum and the worst for those with extensive disseminated disease (89% and 99% vs. 0% and 67%, respectively) [15]. Furthermore, a supratentorial midline location was an unfavorable factor for event-free survival (EFS) [5]. pLGGs involving the optic tract (OPGs), those with multifocal tumors, or those with evidence of dissemination had a higher recurrence rate, although this was not statistically significant [31].

#### 3.1.3. Description of Studies According to Extent of Tumor Resection

**Figure 2:**
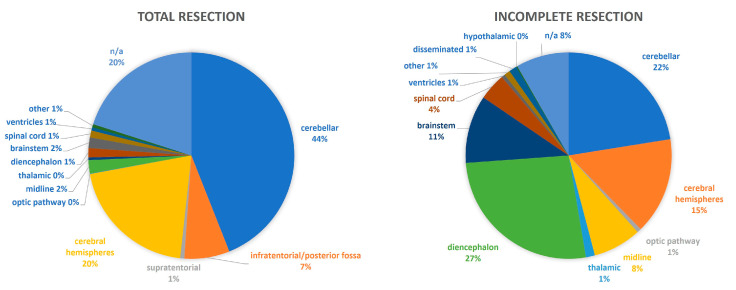
The extent of resection in regard to the tumor location.

Seventeen and fifteen studies analyzed the recurrence of pLGGs with GTR and the progression in those with incomplete resection. The findings are summarized in Table 3 [5,15,22,23,24,25,26,28,29,30,31,32,33,34,35,36,37] and Table 4 [5,15,22,23,24,25,26,27,28,31,32,33,34,35,37], respectively. Except for three studies that analyzed over 500 patients with pLGGs [5,15,24], all other publications were based on a low number of patients (range: 13–107 pts). The extent of resection correlated to the location of the tumor, as shown in Figure 2. Generally, patients with incomplete resection showed a statistically significant lower overall and event-free survival, whereas patients with GTR had a lower risk for progression and death [5,15]. In the study with the longest follow-up of pediatric cerebellar astrocytomas, the extent of surgical resection was the only factor affecting recurrence or progression by multivariate analysis (mean follow-up: 18.4 years) [32].

#### 3.1.4. Description of Studies According to Age

Regarding age, the multivariate analysis in Ryall’s study reported the age at diagnosis as a statistically significant predictive factor for progression and death [15], whereas in the HIT-LGG-1996 study, an age of ≥11 years and an age of <1 year were unfavorable factors for OS and PFS following chemotherapy and for PFS following radiotherapy, respectively [5]. Another study showed an older age at diagnosis to be associated with a decreased hazard ratio (HR) of recurrence for patients 16 to 21 yr of age, compared to patients 0 to 5 yr [24].

#### 3.1.5. Description of Studies According to Histology

Eleven studies analyzed patients with all types of pLGG [5,15,22,24,25,26,27,30,31,32,34], six with PAs only [23,28,29,33,36,37], and one with dysembryoplastic neuroepithelial tumors (DNETs) only [35]. The most common histological diagnoses were PAs, LGGs non-other specified (NOS), gangliogliomas/DNETs, and diffuse astrocytomas in 1783, 318, 246, and 53 patients, respectively.

Patients with recurrent or residual cerebellar pLGGs have a benign clinical course [26]. Grade II glioma was a significantly predictive factor in favor of progression and death in univariate but not in multivariate analysis, whereas PA was a strong negative predictor in both [15].

In the six studies including PAs only, 55 out of 326 patients (16.9%) relapsed or progressed (range 4.4–27.5%) irrespective of the extent of resection [23,28,29,33,36,37]. Two studies analyzed patients with PAs and GTR and found a relapse rate of 13.1% [29,36] with a mean duration of recurrence of 6.4 [25] and 23 months [36], respectively. Notably in one study, all patients who relapsed were asymptomatic [36]. Nodular enhancement on MRI at 3–6 months was significantly associated with recurrence in both univariate and multivariate analyses, although 3 out of 13 patients with nodular enhancement in the first surveillance MRI at 3–6 months regressed [29].

In a study including patients with DNETs only, tumor progression was observed in 3 out of 15 incompletely resected tumors. Notably, the reappearance of seizures was correlated with tumor progression/recurrence [35].

#### 3.1.6. Description of Studies According to Molecular Findings

Only two studies provided molecular data [15,22]. Benes et al. reported on the BRAF status in thalamic and thalamopeduncular pLGGs (10/17 KIAA1549-BRAF fusion, 3/17 BRAF V600E mutation), of which 2 out 10 KIAA1549-BRAF fused patients and one out of three BRAF V600E mutant patients progressed, all without previous additional (systemic) treatment [22].

The largest series on molecular data reported by Ryall et al. included 1037 patients with pLGGs and proposed the stratification of patients in risk groups based on molecular analysis [15]. The low-risk group comprised tumors with gene fusions or germline NF1 mutations, which progress less frequently and eventually stop growing, resulting in very few progressions after 10 years and almost no deaths at 20 years follow-up (10-year PFS of 67% and OS of 98%; 20-year PFS and OS of 58% and 96%, respectively). For these tumors, conservative management seems to be justifiable. The intermediate-risk group comprised tumors with *BRAFp.V600E* without *CDKN2A* deletion, *FGFR1 SNV, IDH1 p.R132H*, or *MET* mutations, with a 10-year PFS and OS of 35% and 90%, respectively. These tumors not only continue to progress with a 20-year PFS of 27% and 20-year OS of 81% but also have a propensity to acquire additional molecular alterations. Thus, these patients may need multiple lines of treatment and longer follow-up. The high-risk group consisted of tumors with *H3.3 p.K27M*, or *BRAF p.V600E* with *CDKN2A* deletion [26], which invariably progress (10-year PFS of 0%), and these patients often succumb to their disease (10-year OS of 41%). Patients with *H3.3 p.K27M—*as expected—do worse than those with *BRAF p.V600E* and *CDKN2A* deletion (10-year PFS and OS of 0% and 35% and 0% and 60%, respectively), but both do much worse than low- and intermediate-risk patients. It should be highlighted though, that according to the current WHO 2021 CNS classification, tumors that were formerly histologically classified as pLGGs—more often PAs—harboring *H3.3 p.K27M* mutations cannot be classified as pLGGs anymore [40]. Furthermore, pLGGs with an undetermined molecular alteration (and hence risk category) showed PFS and OS trends consistent with the representation of both low and intermediate risk (10-year PFS and OS of 51% and 92%, respectively, and 20-year PFS and OS of 34% and 89%). Finally, in the same study, it was shown that tumors with rearrangements had a better prognosis than single nucleotide variation-driven alterations both for progression and death [15]. These different molecular biological features can help to stratify children in risk groups for recurrence which can help to define risk groups for the frequency, interval, and duration of surveillance imaging.

#### 3.1.7. Description of Studies According to Radiological Findings

Only five studies reported on the association of specific radiological findings with progression/recurrence [25,28,29,30,31]. It has been proposed that two consecutive MRIs that test negative for a residual tumor spaced ≥ 3 months apart correspond to a very low likelihood of recurrence in cerebellar PAs, and thus may serve as a good point for the transition to a less frequent surveillance imaging protocol [28]. Contrast enhancement was reported to be present with varying degrees without increasing signal abnormality in 2% of pLGGs of WHO grade 1, but was not considered helpful enough to guide multidisciplinary team decisions in most cases [25]. Nodular enhancement on MRI at 3–6 months was significantly associated with the recurrence of PAs in both univariate (*p* < 0.0001) and multivariate (*p* = 0.0015) analyses [29]. Nodular FLAIR signal in the tumor cavity on the immediate postoperative MRI persisting to the first interval postoperative MRI has been proposed as a useful indication for recurrence or residual disease [30], even though there have been reported regressing cases on subsequent imaging [29]. Lastly, tumor progression was associated with either homogeneous or patchy enhancement on a T1-weighted post-gadolinium scan [31].

### 3.2. Time of Recurrence

Five studies with pLGGs [28,29,34,35,36] and four studies with PAs only [27,32,33,36] reported the mean time of relapse or progression. For all pLGGs, the recurrence time was 27.3 to 59.7 months (range: 9 days to 161.7 months), whereas for PAs specifically, recurrence ranged from 6 months to 48.2 months with the vast majority being observed within the first 12 months. Of notice, only 13% of the pLGGs cases recurred ≥ 5 years postoperatively [31].

For cerebellar astrocytomas, a 28.7% tumor recurrence has been reported within a mean duration of 34.8 months (range: 2 to 132 months). The mean interval to progression was larger for patients with GTR compared to those with radiological residual tumors (59.7 vs. 30.7 months). Additionally, it was reported that 58.6% of all patients recurred/progressed within 2 years after surgery, 13.8% from year 2 to 4, 10.3% from year 4 to 6, and 13.8% from year 6 to 8 [32].

**Figure 3:**
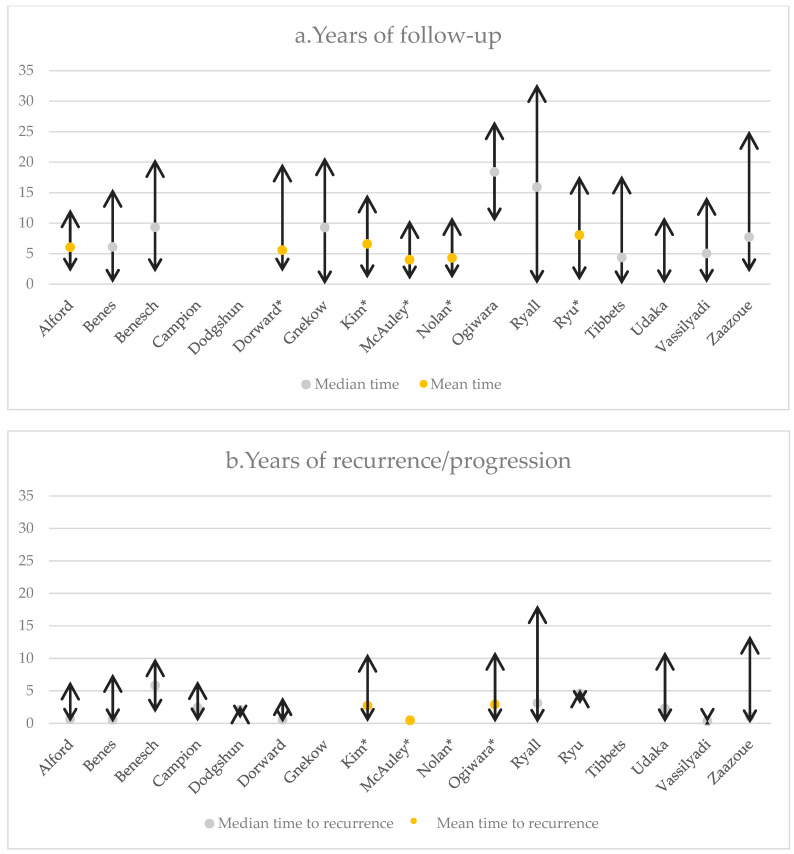
(**a**) The follow-up and (**b**) time to recurrence of the included studies. *: mean time.

Zaazoue et al., in a study of 517 patients, reported radiological evidence of tumor recurrence/progression in 56.5% of patients within a median time of 12.7 months (range: 9 days to 161.7 months). This study observed 63.7% of the recurrences within the first 2 postoperative years, 90.8% by year 5, and 93.2% by year 6 [24]. An overview of the recurrence times also related to the total time of follow-up has been given in Figure 3.

### 3.3. Presence of Symptoms at Diagnosis and at Recurrence/Progression

Descriptions of symptoms at diagnosis and recurrence were analyzed in 4 [24,28,33,35] and 11 [22,23,24,25,26,27,29,30,31,36,37] studies, respectively. Within these datasets, signs of increased intracranial pressure such as headache, nausea, vomiting, and ataxia were the most common symptoms at diagnosis [24,28,33,35]. Interestingly, out of the 270 patients who relapsed/progressed on regular MRI surveillance, 212 patients were asymptomatic. Gnekow et al. reported diencephalic syndrome (DS) at initial diagnosis as an unfavorable factor for PFS and OS [5].

As mentioned in the study with DNETs only, the reappearance of seizures was correlated with the progression/recurrence of the tumor which was proposed as a clinical indicator of a need for further imaging [35].

### 3.4. Regression

The regression of pLGGs was reported in five studies [27,28,29,32,34]. The spontaneous regression of residual cerebellar PAs ranged from 29.6 to 45.5% with a mean duration of 11.9 to 32 months postoperatively (range: 6–50 months) [27,32]. Regression was also reported in 1 out of 53 patients after 10.2 years of follow-up [28], whereas 3 out of 13 patients with nodular enhancement regressed on the first surveillance MRI at 3–6 months [29].

### 3.5. Malignant Transformation

Zaazoue et al. found a malignant transformation in 3 out of 143 (2.1%) patients with pLGGs that were reoperated on due to tumor progression [24]. The malignant transformation of less than 1% was reported in the HIT-LGG 1996 study [5] and in 14 out of 843 (1.7%) patients in Ryall’s study, of whom 5 were H3K27M mutants, and thus, according to WHO 2021, would not be considered as pLGGs [15].

### 3.6. Proposed Follow-Up Schemes

Based on their findings, seven studies proposed follow-up schemes (Table 5 and see Supplementary Table S1) [23,24,25,27,30,34,36].

### 3.7. Health Economics

Three of the extracted articles, apart from proposing follow-up schemes, studied the cost of imaging surveillance concerning recurrence. Dodgshun et al. estimated the cost of the nine scans performed in the first 5 postoperative years to be USD 5855 per patient or USD 193,218 in total, where one recurrence was detected [36]. Kim et al. estimated the cost per recurrence at 5 years to be USD 104,094 per patient and proposed a decrease in the institutional imaging protocol from 10 to 5 MRI scans for the first 5 years, which would provide a potential cost saving of USD 52,047 per recurrence [30]. Finally, Zaazoue et al. calculated the cost of the institutional 15-image protocol to be USD 25,635 and proposed a less expensive protocol with eight images (USD 13 672) with comparative detection rates for patients with GTR [24].

It should be further reported that the cost per patient was estimated by Dodgshun et al. [36] to be AUD 450 per scan or AUD 584 for a scan performed under GA, whereas the average cost of MRI with or without contrast including professional fees was estimated by Kim et al. [30] and Zaazoue et al. [24] at USD 1709.

## 4. Discussion

Ideally, a surveillance imaging protocol during follow-up should balance the aims regarding patient factors, firstly the detection of early recurrence to improve clinical outcomes, and then a reduction in uncertainty. However, there are disadvantages such as the anxiety caused by repeated exams of patients and their families, and the possible late effects from the repeated use of contrast agents and/or general anesthesia. A regular surveillance schedule should also recognize direct and indirect costs for healthcare systems to reduce unnecessary costs.

As shown in this systematic review, despite being routine practice, surveillance by neuroimaging varies and has no standardized approach, and no internationally adopted guidelines are available. Generally, the timing, frequency, and duration of imaging in surveillance protocols should be based on the tumor type, the time from diagnosis, the metastatic status, previous therapy, and specific risk factors per tumor type. In particular, the imaging approach for pLGGs requires not only one single approach but must take additional considerations into account based on their varying epidemiological and histopathological characteristics, biological behavior, clinical consequences such as vision effects, and treatment options. Furthermore, the evolving era in molecular biology and targeted therapies underpins several additional challenges in proposing optimal MRI sequences for imaging surveillance schedules.

One important finding of this systematic review is the significant variation in the definitions of GTR and subtotal resection. To our knowledge, the most detailed recommendation on definitions of the extent of resection based on both radiological and surgical judgment was reported by Gnekow et al. on behalf of the Brain Tumors subcommittee for the reporting of trials in 1995 [39], whereas the recommendations on response assessment in pLGGs from the Response Assessment Pediatric Neuro-Oncology (RAPNO) working group provide only a definition of progressive disease and response for clinical trials [41]. Similar variation in definitions was noticed for the recurrence or progression of pLGGs. The wide variety of definitions does not allow for the comparison of results between different studies. Thus, unifying definitions to be used globally in upcoming trials is extremely important and is necessary to permit the extraction of results for specific pLGG subgroups.

The most frequently studied factor to predict recurrence/relapse in the publications included in this review was the extent of resection. GTR appeared to be associated with a lower likelihood of recurrence [3,16,33,42,43,44,45,46,47,48,49,50]. The reported rate of progression in pLGGs after GTR ranged from 1.7% to 13% [44,47,49,50,51] versus 45.4 to 71.6% after STR [47,50,51]. In this context, several researchers proposed a less intensified protocol for completely resected tumors, especially in cerebellar tumors and/or PAs [23,28,30,35,45], although others disagree [29,32]. Furthermore, a different relative risk of progression has been proposed for patients with <1.5 cm^3^ and ≥1.5 cm^3^ of residual tumor compared to those without (RR 6.0 and 7.9, respectively, *p* < 0.001) [44]. Similar findings of an increasing risk of disease progression with larger volumes of residual tumors have been previously reported [51]. Finally, the recent study by Thomale et al., of 1271 patients with pLGGs and at least one neurosurgical intervention ranging from biopsy to GTR reported that almost 26% of patients required a second surgical intervention, with the hazard of receiving the subsequent surgery being higher for patients with initial biopsy vs. partial vs. subtotal vs. GTR (HR 6.17, 5.65, and 2.69, respectively) [52].

The second most frequently studied factor in this review was tumor location. Cerebellar pLGGs showed a lower recurrence likelihood compared to non-cerebellar pLGGs, especially in the case of GTR [3,16,47,52,53,54]. In fact, a low incidence of glioma-related death and an increased risk of cancer-specific death in children with non-cerebellar tumors has been reported in 4040 adult survivors diagnosed with pLGGS [3]. Furthermore, hemispheric tumors recur less frequently than those in the midline [45]. Generally, brain stem involvement carries a worse prognosis [55]. Suprasellar [47] or hypothalamic–chiasmatic [16,48] location correlated with higher rates of progression or recurrence. Finally, more patients with supratentorial midline PAs suffered two or more progression events, and 75% of patients with less than three progression events had supratentorial midline tumors [56]. Tumors in these locations are harder to resect, and thus, GTR is unlikely, which likely explains the higher progression rates.

Similarly, a younger age at diagnosis was correlated with an increased likelihood of progression or recurrence [47]. Children less than 1 year [48], 2 years [3], 3 years [48,57], or 5 years of age [44] show a worse PFS and OS, although there are researchers who reported no effect of age [51]. A multivariate analysis in the CCLG CNS9702 cohort demonstrated a significantly increased risk for progression for the age group of <1 and 1–5 years compared to the age group older than 10 years (adjusted HR:1.74, 95% CI:1.11–2.73) [16]. Recently, Thomale et al. found that the proportion of patients with more than one surgery decreased with increasing age, with a mean number of surgeries declining from 1.68 in ages less than 1 year, to 1.50 in 1 to less than 3 years, 1.36 in 3 to less than 7 years, 1.33 in 7 to less than 12 years, and 1.26 in those over 12 years [52].

Histologically, PAs have better OS and PFS compared to other histological types [3,51], whereas pilomyxoid astrocytomas have a dismal prognosis in both PFS and OS [16,48,52].

Regarding molecular features, patients with KIAA1549-BRAF fusion have better outcomes, especially when the tumor is cerebellar, and those with FGFR1 mutations—especially those with FGFR1 pK656E point mutation—do worse, although the last study included a small number of both pediatric and adult LGGs [58]. A study with supratentorial midline PAs revealed, apart from the tendency of multiple progressions, a greater occurrence of non-BRAF fusion alterations (BRAFV600E, BRAFD594G, FGFR1, and PTPN11), and other (e.g., KMT2C, CDH1) genes, and also other multiple oncogenic mutations with secondary mutations in either PTPN11 or CDH1 [56]. BRAF V600E in pLGGs is associated with a worse OS, the tendency of multiple progressions, and late deaths related to tumor progression, even at 25 years of follow-up [59]. Moreover, the combination of BRAF V600E and CDKN2A deletion predicted recurrence with an HR of 3.2 [59].

Interestingly, a higher incidence of progression or recurrence for tumors with an exophytic component has been described with the mean interval between the initial surgery and recurrence or progression being double for the patients without these radiological findings [47].

The time of recurrence differs among different publications (median: 1.7–2.5 years, range: 0.36 months to 14.1 years) with most relapses occurring before the third year [49,50] or before year 5–6 [46,52,60] and only a few relapses after 10 years and more [45,60]. The highest recurrence rate in the first year following resection necessitates more aggressive and frequent surveillance imaging during that time, which can be tapered afterward [31]. It should be noted that the recurrence of cerebellar PAs has been reported even 36 and 45 years post-surgical resection [61,62]. Most recurrences were identified only by imaging in asymptomatic patients, whereas up to 35% were detected by clinical symptoms [60].

The regression of residual tumors ranges from 24% to 35.7%, with a mean duration of 21–32 months [50,51]. Thus, some propose a watch-and-wait policy before the second resection is considered unless it is found on the immediate postoperative MRI and is considered safely and easily resectable [53]. Bandyopadhyay et al. highlighted that pLGGs are very unlikely to undergo malignant transformation [3], supporting a wait-and-see strategy as a safe option.

Regarding surveillance protocols, researchers propose performing six to nine MRI scans within a time frame ranging from postoperative to 5 to 10 years, as shown in our results. Studies not included in this review proposed slightly different surveillance schemes, such as one differentiating between children with GTR and those with residual disease (0.5, 1, 2, 3.5, and 5 years for the former, 6-monthly for the first 3 years, then at 4 years, 5 years, and biennially afterward for the latter) [50]. Others are more conservative, proposing 5–10 year intervals even after 5 years of EFS in totally resected cerebellar tumors [53] or a follow-up of 8–10 years in completely resected cerebellar astrocytomas [32]. This contrasts with the opinion that there is no benefit from routine surveillance in these patients, as the likelihood of recurrence is low [49]. Stevens et al., in a review of seven retrospective cases, highlighted the lack of evidence to provide best practice protocol regarding the frequency and duration of surveillance in pLGGs [63] which is in concordance with the findings of the current review.

One of the main limitations of the current review is the lack of consensus regarding the definition of relapse or progression and the extent of resection of pLGGs. Furthermore, it should be acknowledged that there are several subgroups of pLGG mainly determined by their location and how amenable to surgery they are. Thus, different patient populations may have different starting points such as the extent of resection, and there may also be different aims for tumor surveillance. Generally, patients begin with tumor surgery aiming at a maximal safe resection. If GTR or STR is achieved, the main goal of surveillance is to detect relapse or progression and determine an indication for new treatment, surgical or other. Less commonly, patients with only a biopsy or a minimal resection might start non-surgical therapy or may be observed if not symptomatic. Their surveillance aims to detect progression before the patient becomes symptomatic and initiate treatment to preserve neurological or ophthalmological function. Additionally, there are specific groups of patients, most commonly NF1 patients, with pLGGs requiring different approaches due to their distinct characteristics. Finally, some of the reviewed articles addressed directly the risk for relapse/progression and the correlation with specific factors, such as surgery only and/or location, whereas others focused on the natural history of pLGGs.

The strength of this systematic review includes a comprehensive approach of the screening the titles and abstracts, the full texts by two independent reviewers, and the final decision by an additional independent reviewer in case of disagreement. A detailed quality assessment of the included studies was performed, ensuring reliability and relevance. The limitations of this review are linked to the data provided in the included studies. The available data did not allow us to carry out a meta-analysis. The results are therefore limited to descriptive analyses only. We further could not consider molecular subtypes of pLGG in detail as this information was unavailable.

## 5. Conclusions

There is growing evidence that recurrence or progression in pLGGs may be diagnosed several years after diagnosis, with late recurrences occurring even later than 10 years. The evolving molecular era may allow the identification of specific subgroups with the need for more or less intensive surveillance alongside targeted therapy, which is expected to improve outcomes. Thus, to recognize the optimal frequency, intervals, and duration of imaging surveillance, there is an emerging need for an in-depth study of the various characteristics of relapsed or progressed pLGGs in upcoming trials and for routine clinical practice, with unification and an update of the terms “extent of resection” and “progression/relapse” being a prerequisite. Until then, asymptomatic patients with total resection, cerebellar location, PAs, and 5 years post-diagnosis may be considered low-risk for recurrence. Patients with residual disease have a higher chance of progression, for which more frequent and longer FU is advised.

## Figures and Tables

**Table 1:** Definition of extent of resection.

Author Imaging Modality	Gross Total Resection (GTR)		Incomplete Resection		Time of First Postoperative MRI	Comments
Alford et al., 2016 [28] By MRI	GTR/no residual tumor		Subtotal resection		Within the first 48 h	Suspected post-surgical changes/indeterminate
Benes et al., 2022 [22] By MRI	GTR/no residual tumor		Near-total resection: 95–99% resection Subtotal resection: 80–95% resection Partial resection: less than 80% resection		No later than 48 h	No comments
Benesch et al., 2006 [26] By the neurosurgeon’s report and MRI	According to the recommendation of the Brain Tumors subcommittee for the reporting of trials, Gnekow et al., 1995 [39]					
	S1: Total resection, no recognizable residues	R1: No visible tumor on early postoperative CT or MRI without and with contrast enhancement	S2: Remaining tumor of less than 1.5 cc in size, possible localized invasion S3: Remaining tumor of more than 1.5 cc S4: biopsy	R2: Rim enhancement at the operation site only R3: Residual tumor of a measurable size R4: No significant change to preoperative tumor size	Immediate *	No comments
Campion et al., 2021 [25] By MRI	Complete		Incomplete		Usually performed within 48 h of surgery	No further definition
Dodgshun et al., 2016 [36] By MRI	GTR as the absence of enhancing nodular elements				Immediate *	No comments
Dorward et al., 2010 [29] By MRI	GTR as the lack of nodular enhancement on early (within 48 h of surgery) postoperative MRI				Within the first 48 h	No comments
Gnekow et al., 2012 [5] By MRI	Complete		Partial resection		Not mentioned	No further definitions
Gunny et al., 2005 [27] By MRI	The absence of a residual tumor on postoperative MRI regardless of the surgeon’s assessment of the extent of resection		The presence of a residual contrast-enhancing mass or nodule on the earliest postoperative imaging and which correlated with the tumor seen on the original scan.		3–6 months postoperatively	Linear enhancement at resection margins, which may be seen immediately postoperatively and may persist for several years following surgery, was not considered to represent residual disease.
Kim et al., 2014 [30] By MRI	GTR defined as a lack of nodular enhancement on imaging performed in the immediate postoperative period				Within 3 days post-surgery	Patients with a residual tumor on immediate postoperative MRI and who underwent a second craniotomy during the same admission were considered as having GTR if the second postoperative image demonstrated no residual tumor.
McAuley et al., 2019 [23] By MRI	Complete		Partial		Intraoperative or immediate * postoperative MRI scans	No further definitions
Nolan et al., 2004 [35] By MRI	GTR				Not mentioned	No further definitions
Ogiwara et al., 2012 [32] By MRI and neurosurgeon	Group A: Total resection Group B: Total resection by operative observation with radiological residual tumor		Group C: Subtotal resection (Residual tumor < 1 cm^3^)		Within the first 48 h	No comments
Ryall et al., 2020 [15]	GTR		No GTR		Not mentioned	No further definitions
Ryu et al., 2015 [37] Primarily by neurosurgeon’s judgement	GTR: 100% tumor resection		Subtotal resection: ≥50% and <100% Partial resection: <50%		Within 3 months	Percentages of macroscopic resection
Tibbetts et al., 2009 [33] By MRI	GTR: No residual enhancement		Biopsy: <70% resection Subtotal resection: >70% resection with residual solid tumor Near total resection: Linear residual enhancement		Not mentioned	No comments
Udaka et al., 2013 [31] By MRI	GTR		Subtotal resection/biopsy		After surgery *	No further definitions
Vassilyadi et al., 2009 [34] By MRI	Total		Subtotal		On the day following the operation	No further definitions
Zaazoue et al., 2020 [24] By neurosurgeon and MRI	GTR: No evidence of a residual tumor according to the surgeon’s operative notes and the immediate postoperative MRI		Near total resection: no evidence of a residual tumor according to the surgeon’s operative notes and the immediate postoperative MRI Subtotal resection: visible residual tumor recorded following resection surgery Biopsy		Intraoperatively or within 48 h after surgery	No comments

Abbreviations: GTR = gross total resection; MRI = Magnetic Resonance Imaging. * No definition of exact timing postoperatively.

**Table 2:** Definition of progression/relapse.

Year	Tumor Progression	Tumor Relapse	Comments	Author
2004	Progression or relapse		No further definitions	Nolan [35]
2004	An increase in tumor volume observed on serial scans		Spontaneous regression is defined as a reduction in tumor volume in the absence of further surgery or adjuvant radiotherapy	Gunny [27]
2006	Progression and/or relapse is defined as a more than 25% increase in the tumor size radiographically or the emergence of new lesions or CSF positivity.		According to the recommendation of the Brain Tumors subcommittee for the reporting of trials, Gnekow et al., 1995 [39]	Benesch [26]
2009	An increase in the size of the residual tumor after initial treatment	Evidence of a new tumor on neuroimaging when none had been identified on prior post-treatment scans	No comments	Tibbetts [33]
2009	Residual lesion enlargement in all three dimensions compared to the previous study		No comments	Vassilyadi [34]
2010		Recurrence is defined as the development of progressive nodular enhancement on 2 successive follow-up images	No comments	Dorward [29]
2012	Progression	Relapse	No further definitions	Gnekow [5]
2012		Recurrence is based on the evolution of radiological signs with the new appearance of or an increase in contrast enhancement	Definition copied from the article	Ogiwara [32]
2013	Progression	Recurrence	No further definitions	Udaka [31]
2014			No further definitions	Kim [30]
2015	An increase in size over that of the original residual volume	Development of new lesions	Stable disease is defined as no change in tumor size	Ryu [37]
2016	Disease recurrence		No further definitions	Dodgshun [36]
2016	Progression, regression, or stability		If the initial postoperative study demonstrated suspected postsurgical changes or was indeterminate, patients’ imaging records were followed until patients could be categorized as “tumor progression” or “no residual tumor” by the radiology report	Alford [28]
2019	Progression or relapse		No further definitions	McAuley [23]
2019	Progression	Recurrence	No further definitions	Zaazoue [24]
2020	Progression		No further definitions	Ryall [15]
2021	Progression or recurrence is defined as a change in imaging features on surveillance imaging that leads to a change in clinical management		Included in the review as it adds change of clinical management as a factor regarding surveillance	Campion [25]

**Table 3:** Gross total resection vs. recurrence (data order presented according to tumor location).

Author	Years	Institute	No. Pts	Location	Histology ^$^	Years of Follow-Up Mean (Range)	No. of Recurrence (%)	Time to Recurrence
**Alford** [28]	2000–2013	Texas, USA	41	41 cerebellar	41 pilocytic astrocytoma	(0.3–6.4)	6 (15)	range: 0.3–6.4 y (median 0.6 y)
**Benesch** [26]	1983–2003	Austria	18	18 cerebellar	15 pilocytic astrocytoma 3 fibrillary astrocytoma	median 9.3 (2–20.5)	2 (13), both pilocytic astrocytoma 1 patient died postoperatively	one patient: 10 y one patient: 20 m
**McAuley** [23]	2007–2017	Liverpool, UK	36	36 cerebellar	36 pilocytic astrocytoma	4 (0.8–10.5)	1 (3)	1 at 2.2 y
**Ogiwara** [32]	1983–1999	Chicago, USA	51	51 cerebellar	entire cohort: 55 pilocytic astrocytoma 43 low-grade astrocytoma nos 2 fibrillary astrocytoma 1 grade II astrocytoma nos	18.4 (10.3–26.7)	3 (6)	mean 60 m
**Ryu** [37]	1995–2013	Republic of Korea	11	11 cerebellar	11 pilocytic astrocytoma	8 (0.6–17.8)	0 (0)	none
**Vassilyadi** [34]	1987–2007	Ottawa, Canada	19	19 cerebellar	11 pilocytic astrocytoma 8 non-pilocytic astrocytoma	7 (0.2–14.3)	0 (0)	none
**Dorward ^#^;** [29]	n/a	St Louis, USA	40	40 infratentorial	40 pilocytic astrocytoma	5.6 (2.1–19.8)	11 (28)	range: 2–48.2 m (median 6.4 m, mean 16 m) 10 at scans 3–6 m 1 at 48.2 m
**Dodgshun** [36]	1996–2013	Australia	67	58 posterior fossa 9 supratentorial	67 pilocytic astrocytoma	at least 5 in 33 patients	3 (4)	range: 9–33 m
**Campion** [25]	2007–2013	London, UK	33	25 cerebellar 1 optic pathway 7 “other” (thalamic, midbrain, lobar)	entire cohort: 63 pilocytic astrocytoma 3 pleomorphic xanthoastrocytoma 1 angiocentric glioma	n/a	1 cerebellar 3 “other” optic pathway not classified	13 m unknown unknown
**Gnekow** [5]	1996–2004	Germany	343	96 cerebral hemisphere 29 midline supratentorial 188 cerebellum 15 brainstem 8 spinal cord 7 lateral ventricles	entire observation arm: 455 pilocytic astrocytoma 77 ganglioglioma/dysembryoplastic neuroepithelial tumor/other mixed glioneuronal tumors 33 diffuse astrocytoma 14 subependymal giant cell astrocytoma 12 pleomoprhic xanthoastrocytoma 7 oligodendroglioma 6 low grade glioma nos 4 oligoastrocytoma 60 unclear/no histology	entire cohort: median 9.3 (0–20.8)	54 (15)	n/a
**Kim ^^^;** [30]	1993–2003	Boston, USA	67	41 cerebellar 16 temporal 4 parietal 2 frontal 2 brainstem 2 occipital	46 pilocytic astrocytoma 14 ganglioglioma 6 dysembryoplastic neuroepithelial tumor 1 glioneuronal tumor	6.6 (1–14.7)	13 (19)	4 in the first 6 m 3 in 6–12 m 5 in 3–5 y 1 in 10 y
Ryall *; [15]	1986–2017	Toronto, Canada	365	153 hemispheric 20 diencephalon 6 brainstem 179 cerebellum 7 spine	140 pilocytic astrocytoma 106 low-grade glioma nos 41 ganglioglioma 22 dysembryoplastic epithelial tumor 20 diffuse astrocytoma 11 oligodendroglioma 10 glioneuronal tumor 5 angiocentric glioma 8 pleomorphic xanthoastrocytoma 2 sesmoplastic infantile ganglioglioma	15 (0.1–32.8)	41 (and 13 unknown outcomes) (11)	0.1–18.2 y, median 3.1 y 5 patients >10 y
**Tibbetts ^#^;** [33]	1990–2004	St Louis, USA	73	entire cohort: 51 cerebellar 15 brain stem 2 spinal cord 25 supratentorial 12 optic pathway	73 pilocytic astrocytoma	median entire cohort: 4.4 (0–17.8)	12 (16)	
**Benes** [22]	2005–2020	Czech Republic	6	6 thalamus/thalamopeduncular	5 pilocytic astrocytoma 1 grade 2 glioma (?) nos	4.7 (1.9–8.5)	0 (0)	none
Nolan *; [35]	1993–2002	Toronto, Canada	9	total cohort: 10 temporal 8 frontal 6 parietal 2 occipital	9 dysembryoplastic neuroepithelial tumor	entire cohort: 4.3 (1–11)	0 (0)	none
**Udaka** [31]	1994–2010	San Diego, USA	38	n/a	n/a	2 m–11 y	9 (24)	0–60 m
**Zaazoue ^^^;** [24]	1990–2016	Boston, USA	240	n/a	n/a	(2–25.1)	84 (35)	median time to recurrence complete cohort: 12.7 m (range: 9 d–161.7 m) 63.7% of recurrences within the first 2 yr postoperatively, 90.8% by 5 yr, and 93.2% by 6 yr)

Abbreviations: n/a = not available; nos = not otherwise classified; m = months; y = year. ^#^ Data from the paper from Dorward may be included in the paper from Tibbetts. ^ Data from the papers from Kim and Zaazoue overlap. * Data from the paper from Nolan are included in the paper from Ryall. ^$^ For some papers, the histology data could not be extracted for just the patients with GTR; thus, the entire cohort is mentioned.

**Table 4:** Partial resection vs. progression (data order presented according to tumor location).

Author	Years	Place	No. Patients	Location	Histology ^$^	Years of FU Mean (Range)	No. of Patients with Progression (%)	Time to Progression	Additional Therapy
**Alford** [28]	2000–2013	Texas, USA	12	12 cerebellar	12 pilocytic astrocytoma	(0.3–6.4)	4 (33)	median 5 m	3 immediate: 2 re-resection 1 focused radiation
**Benesch** [26]	1983–2003	Austria	9	9 cerebellar	6 pilocytic astrocytoma 2 fibrillary astrocytoma 1 mixed hamartoma/pilocytic astrocytoma	(2–20.5) Median 9.3 y	1 (11)	3 y 1 died due to brain stem infiltration/compression	3 immediate radiotherapy (one died) 1 re-resection 3 y after diagnosis
**Gunny** [27]	1988–1998	London, UK	11	11 cerebellar	10 pilocytic astrocytoma 1 fibrillary astrocytoma	6.8 (2–13.3)	5 (45)	progression at 7, 9, 12, 13, and 20 months (4 PA, 1 FA) 5 regression (PA)	1 re-resection 1 radiotherapy 3 re-resection + radiotherapy
**McAuley** [23]	2007–2017	Liverpool, UK	4	4 cerebellar	4 pilocytic astrocytoma	4 (0.8–10.5)	4 (100)	5.4 m, all < 12 m	re-resection
**Ogiwara** [32]	1983–1999	Chicago, USA	50	50 cerebellar	entire cohort: 55 pilocytic astrocytoma 43 low-grade astrocytoma noc 2 fibrillary astrocytoma 1 grade II astrocytoma noc	18.4 (10.3–26.7)	26 (52)	30.7 m (0–132 m)	n/a
**Vassilyadi** [34]	1987–2007	Ottowa, Canada	9	9 cerebellar	5 non-pilocytic astrocytoma 4 pilocytic astrocytoma	4.4 (0.8–7.8)	1 non- pilocytic astrocytoma (2) 1 pilocytic astrocytoma (25)	5 m 3 m	stable disease but 2nd surgery: 4 2nd surgery after progression: 2
**Campion** [25]	2007–2013	London, UK	29	15 cerebellar 4 optic pathway 10 “other” (thalamic, midbrain, lobar)	entire cohort: 63 pilocytic astrocytoma 3 pleomorphic xanthoastrocytomas 1 angiocentric glioma		7 cerebellar (47)	mean 26 m (4–46 m)	
							3 optic pathway (75)	unknown	
							6 other (60)	unknown	
**Gnekow** [5]	1996–2004	Germany	271	60 cerebral hemisphere 80 midline supratentorial 77 cerebellar 34 brainstem 14 spinal cord 6 lateral ventricles	entire observation arm: 455 pilocytic astrocytoma 77 ganglioglioma/dysembryoplastic neuroepithelial tumor/other mixed glioneuronal tumors 33 diffuse astrocytoma 14 subependymal giant cell astrocytoma 12 pleomoprhic xanthoastrocytoma 7 oligodendroglioma 6 low-grade glioma nos 4 oligoastrocytoma 60 unclear/no histology	entire cohort: median 9.3 y (0–20.8 y)	292 (59)	n/a	49 continued observation 76 resection 99 chemotherapy # 80 radiotherapy # # 12 of these were patients with complete resection but relapse
Ryall *; [15]	1986–2017	Toronto, Canada	538	85 hemispheric 281 diencepalon 77 brainstem 51 cerebellar 31 spine 13 disseminated	163 pilocytic astrocytoma 154 LGG NOS 108 NF 38 ganglioglioma 30 diffuse astrocytoma 14 dysembryoplastic neuroepithelial tumor 11 glioneuronal tumor 10 oligodendroglioma 7 pleomorphic xanthoastrocytoma 2 angiocentric glioma1 desmoplastic infantile astrocytoma	15.0 (0.2–32.6)	243 (and 12 unknown) (45)	mean 5.5 y, median 4.3 y (0.1–18.9)	entire cohort: 348 chemotherapy 31 targeted inhibitor 176 radiation therapy NB: part upfront, part at progression
**Tibbetts** [33]	1990–2004	St Louis, USA	34	entire cohort: 51 cerebellar 15 brain stem 2 spinal cord 25 supratentorial 12 optic pathway	34 pilocytic astrocytoma	median entire cohort: 4.4 y (0–17.8)	11 (32)	n/a	entire cohort (time point): 13 resection and chemotherapy 18 resection and radiation
**Benes** [22]	2005–2020	Czech Republic	15	15 thalamus/thalamopeduncular	11 pilocytic astrocytoma 4 grade 2 nos	6.5 (0.3–15.6)	9 (60)	9 (2.0 m–91.1 m)	2 observation 2 surgery 2 chemotherapy 3 surgery + chemotherapy
Nolan * [35]	1993–2002	Toronto, Canada	15	entire cohort: 10 temporal 8 frontal 6 parietal 2 occipital	15 dysembryoplastic neuroepithelial tumor	entire cohort: 4.3 (1–11)	3 (20)	12–18 m in 2 5 y in 1	7 with second and 2 with third excision due to refractory seizures
**Ryu** [37]	1995–2013	Republic of Korea	8	3 OPG+ hypothalamus 2 hypothalamus 2 brainstem 1 temporal lobe	8 pilocytic astrocytoma	8 (0.6–17.8)	3 (38)	3.0, 4.6 (both OPG/HP), 5.2 y (brainstem)	immediate in 6: 3OPG/hypothalamic: 2 chemotherapy, 1 RT 2 hypothalamic: 1 gamma knife, 1 chemotherapy 1 brainstem: radiotherapy
**Udaka** [31]	1994–2010	San Diego, USA	64	n/a	n/a	2 m–11 y	35 (55)	0–>60 m	n/a
**Zaazoue** [24]	1990–2016	Boston, USA	24	n/a	n/a	(2–25.1)	n/a	3 patients with malignant transformation (1 after radiotherapy, 1 after chemotherapy, 1 after both); all died	n/a

OPG = optic pathway glioma; n/a = not applicable; HP = hypothalamic glioma. * Data from the paper from Nolan are included in the paper from Ryall. ^$^ For some papers, the histology data could not be extracted for just the patients with partial resection; thus, the entire cohort is mentioned.

**Table 5:** Proposed time points of the follow-up schemes for patients with pLGGs treated with surgery only.

Months		0	1	3	6	9	12	18	24	30	36	42	48	54	60	66	72	84	108	114	120	Total
Kim ^ [30]	GTR	●		●			●	●														4
Zaazoue ^ [24]	GTR	●		●		●			●		●				●							6
	All	●		●	●		●		●		●				●							7
Dodgshun [36]	GTR	●		3–6			●		●			●			●						●	7
	All																					
Gunny [27]	Cerebellar R+	●			●		●	●	●	●	●		●		●			●	●			11
McAuley [23]	Cerebellar R0 and R+	●			●			●		●												4
Vassilyadi [34]	Cerebellar R0 and R+	●			●			●			●				●							5
Campion [25]	Cerebellar R+	●				●		●		●												4

Abbreviations: GTR gross total resection, R+ with residue, R0 without residue. ^ Cohorts overlap.

## Supplementary Materials

The following supporting information can be downloaded at https://www.mdpi.com/article/10.3390/curroncol31110541/s1, Supplementary Data S1: Search Strategy LGG review; Table S1: Proposed follow-up schemes for patients treated with surgery only.
