# Supplementary material for: Towards a Risk-Based Follow-Up Surveillance Imaging Schedule for Children and Adolescents with Low-Grade Glioma
Source: Curr Oncol. 2024 Nov 18;31(11):7330–51. doi: 10.3390/curroncol31110541 (PMC11592938; doi:10.3390/curroncol31110541)
Supplement: Supplementary file 1 [file curroncol-31-00541-s001.zip › Supplement Data S1 Search strategy LGG review.pdf]

Search strategy LGG review

PICO Framework
Population: childhood, adolescent and young adult cancer patients with low grade glioma
Intervention: Imaging
Comparison: none
Outcome: SMN, progression, relapse, cavernoma, vascular outcomes
(Surveillance)

1. **Population:** CAYA cancer patients (from IGHG)	Infan* OR toddler* OR minors OR minors* OR boy* OR girl* OR kid OR kids OR child* OR Child[MeSH Terms] OR Infant[MeSH Terms] OR schoolchild* OR schoolchild OR school child* OR adolescen* OR Adolescent[MeSH Terms] OR juvenil* OR youth* OR teen* OR under*age* OR pubescen* OR pediatrics[MeSH Terms] OR pediatric* OR paediatric* OR peadiatric* OR school* OR young adult[MeSH Terms] OR young adult
2. **Population:** Low-grade glioma	((Glioma[MeSH Terms] OR glioma* OR Pituitary Neoplasms[MeSH Terms] OR pituitary OR pineal OR Supratentorial Neoplasms[MeSH Terms] OR supratentorial OR Infratentorial Neoplasms[MeSH Terms] OR infratentorial OR Hypothalamic Neoplasms[MeSH Terms] OR Astrocytoma[MeSH Terms] OR Cerebellar Neoplasms[MeSH Terms]) AND ((low-grade glioma*) OR (low grade glioma*) OR LGG)) OR opticus glioma* OR Optic Nerve Glioma[MeSH Terms] OR optic nerve glioma* OR Oligodendroglioma[MeSH Terms] OR oligodendroglioma* OR (pilocytic AND astrocytoma*) OR Ganglioglioma[MeSH Terms] OR ganglioglioma* OR (low-grade AND brain AND tumour) OR (low-grade AND brain AND tumor) OR (incidental* AND (CNS OR brain)) OR (glioneuronal* AND (CNS OR Brain))
3. Combine	#1 AND #2
4. **Intervention:** Imaging	MRI OR (magnetic resonance imaging) OR Magnetic Resonance Imaging[MeSH Terms]
5. Combine	#3 AND #4
6. **Outcome:** SMN (from IGHG)	(Radiation Induced Neoplasm*) OR (Radiation Induced Cancer*) OR Neoplasm, Second Primary[MeSH Terms] OR (Second Primary Neoplasm*) OR (Metachronous Second Primary Neoplasm*) OR (Second Malignanc*) OR (Second Neoplasm*) OR (Metachronous Neoplasm*) OR (Therapy Associated Neoplasm*) OR (Therapy Related Neoplasm*) OR (Treatment Associated Neoplasm*) OR (Treatment Related Neoplasm*) OR (Therapy Associated Cancer*) OR (Therapy Related Cancer*) OR (Treatment Related Cancer*) OR (Treatment Associated Cancer*) OR (Second Primary Cancer*) OR (Second Cancer*) OR Neoplasms, Radiation-Induced/etiology OR Neoplasms, Radiation-Induced OR Neoplasms, Radiation effects OR Neoplasms, Second Primary[MeSH Terms] OR Neoplasms, Second Primary[MeSH Terms] OR (second primary malignancy) OR second primary malignancies OR second malignant neoplasm* OR SMN OR subsequent malignant neoplasm* OR subsequent neoplasm* OR new malignancy OR new malignancies
7. **Outcome:** progression, relapse	Neoplasm Recurrence, Local[MeSH Terms] OR Neoplasm Regression, Spontaneous[MeSH Terms] OR Remission, Spontaneous[MeSH Terms], Neoplasm, Residual[MeSH Terms] OR Disease Progression[MeSH Terms] (((contrast media) OR gadolinium) AND deposit*) OR Disease-Free Survival[MeSH Terms] OR relapse OR progression OR recurrence
8. **Outcome:** vascular outcomes (stroke, bleeds), cavernoma	Brain Infarct [MeSH Terms] OR Hemorrhagic Stroke[MeSH Terms] OR Ischemic Stroke[MeSH Terms] OR Hemangioma, Cavernous, Central Nervous System[MeSH Terms] OR Moyamoya Disease[MeSH Terms] OR stroke OR cavernoma OR moyamoya
9. Combine	#6 OR #7 OR #8

10. Combine	#5 AND #9
11. **Outcome:** Surveillance, follow-up	long term effect* OR long term adverse effects[MeSH Terms] OR follow up stud* OR aftercare [MeSH Terms] OR aftercare* OR after treatment OR Follow-Up Studies[MeSH Terms] OR surveillance
12. Combine	#10 AND #11
13. Humans only	animals[MeSH Terms] NOT humans[MeSH Terms]
14. Combine	#12 NOT #13
15. Date restriction	"2003/01/01"[Date - Publication] : "2023/12/31"[Date - Publication]
16. Combine	#14 AND #15
