# Supplementary material for: Towards a Risk-Based Follow-Up Surveillance Imaging Schedule for Children and Adolescents with Low-Grade Glioma
Source: Curr Oncol. 2024 Nov 18;31(11):7330–51. doi: 10.3390/curroncol31110541 (PMC11592938; doi:10.3390/curroncol31110541)
Supplement: Supplementary file 1 [file curroncol-31-00541-s001.zip › Supplementary Table S1.pdf]

Supplementary material Table S1. Proposed follow-up schemes for patients treated with surgery only.

Author	Population	Scheme
**Campion**	All	Tailor to the location as well as the degree of initial resection. Follow-up scheme of McAuley for cerebellar tumors; more data needed on surveillance patterns in non-cerebellar tumors
**Dodgshun**	Following GTR	6 MRI scans postoperatively: direct postop, 3–6 months, at 1, 2, 3.5, and 5 years
**Gunny**	Cerebellar with residual disease	Postop 24–48 h, 6 m, 12 m, 18 m, 24 m, 30 m, 36 m, 48, 60 m, with residual tumor 2-yearly thereafter
**Kim ^**	Following GTR	Postop 1–3 days, 3 months, 1, 2, 5, and 10 years. A 5-year surveillance schedule beyond 10 years.
**McAuley**	Cerebellar with and without residual disease	Completely resected patients: 6 m, 18 m, and 30 m, then stop
**Vassilyadi**	Cerebellar with and without residual	Postop, 6 m, 18 m, 3 years, and 5 years.
**Zaazoue ^**	All	For all patients, an 8-image surveillance protocol at 0, 3, 6, 12, 24, 36, 60, and 72 m<br>For patients with GTR, a 6-image protocol at 0, 3, 9, 24, 36, and 60 mo ($10,254) is sufficient.

^ Cohorts overlap
